# Maternal Adherence to Healthy Dietary Patterns During Pregnancy and Gestational Weight Gain

**DOI:** 10.3390/nu17162707

**Published:** 2025-08-21

**Authors:** Shan-Xuan Lim, Siona Wadhawan, Elizabeth A. DeVilbiss, Priscilla K. Clayton, Kathryn A. Wagner, Jessica L. Gleason, Zhen Chen, Cuilin Zhang, Katherine L. Grantz, Jagteshwar Grewal

**Affiliations:** 1 Office of the Director, Division of Population Health Research, Division of Intramural Research, *Eunice Kennedy Shriver* National Institute of Child Health and Human Development, 6710B Rockledge Drive, Bethesda, MD 20817, USA; 2 Department of Community Health, School of Arts and Sciences, Tufts University, 574 Boston Avenue, Suite 208, Medford, MA 02155, USA; 3 Department of Biology, School of Arts and Sciences, Tufts University, Robinson Hall, 200 College Avenue, Medford, MA 02155, USA; 4 Department of Biology, School of Arts and Sciences, Tufts University, Robinson Hall, 200 Boston Avenue, Medford, MA 02155, USA; 5 Epidemiology Branch, Division of Population Health Research, Division of Intramural Research, *Eunice Kennedy Shriver* National Institute of Child Health and Human Development, 6710B Rockledge Drive, Bethesda, MD 20187, USA; 6 School of Arts and Sciences, Massachusetts College of Pharmacy and Health Sciences, 179 Longwood Avenue, Boston, MA 02155, USA; 7 Biostatistics and Bioinformatics Branch, Division of Population Health Research, Division of Intramural Research, *Eunice Kennedy Shriver* National Institute of Child Health and Human Development, 6710B Rockledge Drive, Bethesda, MD 20817, USA; 8 Global Centre for Asian Women’s Health, Yong Loo Lin School of Medicine, National University of Singapore, 10 Medical Drive, Singapore 117597, Singapore; 9 Department of Obstetrics & Gynecology, Yong Loo Lin School of Medicine, National University of Singapore, 10 Medical Drive, Singapore 117597, Singapore; 10 Bia-Echo Asia Centre for Reproductive Longevity and Equality (ACRLE), Yong Loo Lin School of Medicine, National University of Singapore, 10 Medical Drive, Singapore 117597, Singapore; 11 Department of Nutrition, Harvard T.H. Chan School of Public Health, 655 Huntington Avenue, Boston, MA 02115, USA

**Keywords:** diet, dietary patterns, food frequency questionnaire (FFQ), pregnancy, gestational weight gain

## Abstract

**Background/Objectives**: Suboptimal gestational weight gain (GWG) has been linked to increased risks of adverse maternal outcomes. Evidence linking diet in pregnancy to GWG remains limited. We assessed relationships between adherence to five dietary patterns (Planetary Health Diet [PHD], Dietary Approaches to Stop Hypertension [DASH], Alternate Mediterranean Diet [AMED], Healthy Eating Index [HEI], and Alternate Healthy Eating Index [AHEI]) and 2009 Institute of Medicine GWG categories. **Methods**: Women expecting singleton pregnancies participated in the NICHD Fetal Growth Studies and completed a food-frequency questionnaire (FFQ) at 8 to 13 weeks of gestation that captured their baseline diet. Adherence to each dietary pattern was calculated, with higher scores indicating greater adherence. Women were categorized into low, moderate or high adherence to each dietary pattern. Using multinomial logistic regression, we estimated adjusted odds ratios and 95% confidence intervals [OR (95% CIs)] of inadequate or excessive GWG (reference category: adequate), for high vs. low adherence to each dietary pattern. **Results**: In the full cohort, women with high vs. low adherence to DASH, AMED, HEI, or AHEI (but not PHD) had a 13% to 31% lowered odds of inadequate total GWG [ranging from 0.87 (0.58, 1.31) for AMED to 0.69 (0.48, 0.99) for DASH]. High adherence to DASH or HEI was associated with lower odds of inadequate first-trimester GWG, after correcting for multiple testing [DASH: 0.36 (0.22, 0.61), HEI: 0.49 (0.30, 0.79)]. No significant association was observed between any of the dietary patterns and excessive total and trimester-specific GWG. **Conclusions**: Greater adherence to several dietary patterns was associated with lowered odds of inadequate GWG. Future studies could characterize these diets objectively by identifying metabolite signatures and examining their associations with GWG.

## 1. Introduction

Optimal GWG is key for both maternal and fetal health [1,2]. GWG above or below existing guidelines (i.e., excessive or inadequate GWG) has been associated with adverse maternal health and offspring growth and neurodevelopment outcomes [3,4]. In the United States, 51% of pregnant women had excessive GWG and 21% had inadequate GWG between 2000 and 2014 [3]. Maternal weight gain during pregnancy is typically attributed to several physiological mechanisms, including the development of the fetoplacental unit; increased maternal blood, extravascular volumes, and breast tissue; and increased maternal accumulation of cellular water, fat, and protein [2]. In 2009, the Institute of Medicine (IOM) (US) and National Research Council (US) Committee to Reexamine IOM Pregnancy Weight Guidelines recognized dietary intake during pregnancy as a key contributor to GWG [1]. Although weight gain is an inherent aspect of pregnancy, studies have unequivocally demonstrated links between inadequate or excessive gestational weight gain and adverse maternal and neonatal outcomes [5]. More recently, the U.S. Departments of Health and Human Services (HHS) and Agriculture (USDA) highlighted nutrition during pregnancy and GWG as a relevant and important scientific consideration for developing the 2025–2030 Dietary Guidelines for Americans (DGA) [6].

In order to provide evidence-based recommendations for developing the DGA, the 2025 Dietary Guidelines Advisory Committee (DGAC) reviewed available evidence and concluded that healthy maternal dietary patterns (rich in vegetables, fruits, legumes, nuts, and fish with lower intakes of added sugars) during pregnancy are associated with lowered risks of excessive GWG [6]. In contrast, the DGAC did not draw any conclusions about the relationship between maternal dietary patterns during pregnancy and inadequate GWG, as the existing evidence was mixed [6]. Further research is needed to better understand the link between maternal dietary patterns during pregnancy and GWG outcomes. Recent statistics indicate that merely 28% of pregnant women meet the IOM criteria for adequate GWG [3]. Therefore, identifying specific dietary patterns that help to promote adequate GWG is worthwhile.

Among the candidates are dietary patterns designed to emphasize healthy food consumption and promote positive health outcomes in non-pregnant populations [7,8]. The Planetary Health Diet (PHD), Alternative Mediterranean Diet (AMED), and Dietary Approaches to Stop Hypertension (DASH) were all developed to reduce risks of non-communicable diseases, but each emphasizes distinctive dietary components. The PHD (which is designed to promote environmental sustainability alongside individual health) strictly limits the intake of animal-sourced foods [9,10]. The AMED encourages the intake of fish and unsaturated oils and is known to reduce the risk of coronary heart disease and stroke [11]. The DASH diet was originally developed to lower blood pressure and encourages low-fat dairy intake [12]. Meanwhile, both the Healthy Eating Index (HEI) and the Alternate Healthy Eating Index (AHEI) align with the Dietary Guidelines for Americans in 2010 [13,14]. The HEI and AHEI are largely similar, but studies have shown that the latter is more strongly associated with reduced risks of coronary heart disease and type II diabetes [14,15,16].

To date, these five dietary patterns have not been collectively examined within a single study, let alone one that evaluates the relationships between these dietary patterns and GWG among pregnant women, with attention to absolute weight gain observed at several timepoints over the course of pregnancy. To address these gaps, we assessed the relationships between adherence to each of the dietary patterns and (1) total GWG based on 2009 IOM GWG categories and (2) trimester-specific GWG. This novel analysis leverages data obtained from a multisite, prospective pregnancy cohort in the US.

## 2. Materials and Methods

### 2.1. Study Design

The *Eunice Kennedy Shriver* National Institute of Child Health and Human Development (NICHD) Fetal Growth Studies recruited pregnant women at 8 and 13 weeks of gestation with singleton pregnancies from 12 clinical sites across the US [17]. This prospective study was initially designed to establish standards for fetal growth. The women enrolled in the study were followed for up to four additional visits during pregnancy [18]. Of the 2802 women originally enrolled in the study, a subset of 1980 women were invited to participate in the nutrition sub-study [19]. The institutional review boards of NICHD and clinical and data coordinating centers approved the study (ClinicalTrials.gov, NCT00912132). Written informed consent was obtained from all women prior to enrollment. Further details of the cohort are described elsewhere [17].

### 2.2. Dietary Assessments

At enrollment, women completed a validated, semi-quantitative modified version of the Diet History Questionnaire-II (DHQ-II) that reflected habitual diet in the prior three months (periconception and early pregnancy) [20,21]. For each woman, nutrient intake and My Pyramid Equivalent Database (MPED) food groups were obtained based on the reported frequency and serving size of the respective DHQ-II food item, using the Diet*Calc software (version 1.4.3) [20,22]. Women with implausible estimated energy intake (<600 or >6000 kcal/day) were excluded (*n* = 86) [19]. Using estimated nutrient intake and MPED food group data, we quantified adherence to five dietary patterns using continuous scores. The Planetary Health Diet Index (PHD) consists of six adequacy and eight moderation components, each scored on a scale from 0 to 10, with a theoretical overall score ranging from 0 (lowest) to 140 (highest) [10]. The AMED consists of 9 components, with a theoretical overall score ranging from 0 (lowest) to 9 (highest) [11]. The DASH consists of 8 components, each scored on a scale from 0 to 5, with a theoretical overall score ranging from 0 (lowest) to 40 (highest) [12]. The HEI consists of 10 components, each scored on a scale from 0 to 10, with a theoretical overall score ranging from 0 (lowest) to 100 (highest) [13]. The AHEI consists of 11 components, each scored on a scale from 0 to 10, with a theoretical overall score of 0 (lowest) to 110 (highest). The PHD, HEI, and AHEI were scored based on meeting target reference values for the respective dietary components. The scoring for AMED and DASH was specific to our study, according to the distribution of each respective dietary component. For each dietary pattern, a higher score is indicative of greater adherence. We further categorized women into tertiles of adherence to each dietary pattern (T1: low; T2: moderate; and T3: high) (Supplementary Table S1).

### 2.3. Gestational Weight Gain (GWG)

Maternal pre-pregnancy body mass index (BMI, kg/m^2^) at enrollment was calculated as a function of self-reported weight (kg) divided by the squared self-reported height (m^2^). Maternal self-reported height and weight were highly correlated to measured height and weight, respectively (r = 0.95 for height; r = 0.97 for weight) [23]. Total gestational weight gain (GWG) was calculated as the difference between the maternal weight at delivery and self-reported pre-pregnancy weight [24]. During pregnancy, maternal pregnancy weights were either measured at each research visit or abstracted from prenatal records. Maternal weight trajectories throughout gestation were modeled using linear mixed models with cubic splines, including gestational ages as fixed effects [25]. Each woman’s trimester-specific GWG was expressed in kilograms (first trimester: up to 13.9 weeks of gestation; second trimester: between 14 and 27.9 weeks of gestation; and third trimester: from 28 weeks to delivery). Based on the 2009 IOM guidelines, which are specific to a woman’s pre-pregnancy BMI [1], we categorized total and trimester-specific GWG (continuous variables) into one of three categories (inadequate, adequate, or excessive).

### 2.4. Covariates

At enrollment, study personnel interviewed women to obtain data on socio-demographic characteristics (including age, marital status, and race/ethnicity) and reproductive history (parity) [17]. Self-reported vegetarianism was defined based on the binary response to the question “For all of the past 3 months, have you followed a vegetarian diet?”. Women independently completed the validated Pregnancy Physical Activity Questionnaire (PPAQ), from which total physical activity (MET-min/week) was calculated for the previous 12 months [26].

### 2.5. Statistical Analysis

Of the 1980 women invited to participate in the nutrition sub-study, we excluded women as described in Supplementary Figure S1, leaving *n* = 1530 in the analytical sample. The baseline characteristics of the included and excluded women were compared using the chi-square test for categorical variables and the one-way Analysis of Variance (ANOVA) or Kruskal–Wallis test for continuous variables. The women included in the analytical sample tended to have more demographic attributes consistent with higher socioeconomic status than those excluded (Supplementary Table S2). The baseline characteristics of women across the tertiles of adherence to each dietary pattern were also compared using appropriate statistical tests. Additionally, we compared intake of macronutrients, micronutrients of importance during pregnancy [27], absolute total GWG (in kilograms), and the distribution of women with adequate total and trimester-specific GWG across the tertiles of adherence to each dietary pattern.

We calculated Spearman correlations [28] between (1) each pair of dietary pattern scores and (2) each dietary pattern score and key food groups of interest (Supplementary Figures S2 and S3, Supplementary Text). We used multinomial logistic regression to assess the relationships between extremes of adherence to each dietary pattern (high vs. low) and inadequate or excessive total and trimester-specific GWG, estimating odds ratios (ORs) and 95% confidence intervals (CIs). For all models, the reference group was women with adequate GWG, with the list of covariates to include determined *a priori*. These covariates included key confounders of interest for the relationship between diet and GWG according to the 2025 DGAC [6], a previous publication on a related topic [25], and were selected using a Directed Acyclic Graph (DAG) (maternal age, race/ethnicity, education, parity, and total physical activity) (Supplementary Figure S4). We conducted several sensitivity analyses: (1) additional adjustment for daily caloric intake [29], (2) additional adjustment for gestational diabetes mellitus (GDM) or hypertensive disorders of pregnancy (HDP) in the current pregnancy, and (3) several stratified analyses were conducted to assess the robustness of our main findings. The three stratified analyses conducted were: limiting the analytical sample to (A) women without gestational diabetes mellitus (GDM) and hypertensive disorders of pregnancy (HDP) (*N* = 1348); (B) women with GDM (*N* = 86); and (C) women with HDP in the current pregnancy (*N* = 105). Given the multiple statistical tests performed, there was an increased risk of type I errors [30]. As such, we presented the unadjusted and adjusted (Benjamini–Hochberg) *p*-values of the main associations to control the false discovery rate [31]. The threshold for significant *p*-values was set at <0.05. R Studio (R version 4.4.0) was used to perform all analyses.

## 3. Results

### 3.1. Study Characteristics

Within the analytical sample of 1530 pregnant women, those with a high adherence to all five dietary patterns were generally older, Asian/Pacific Islander or Non-Hispanic White, nulliparous or primiparous, married or living with a partner, educated at the level of an undergraduate/postgraduate degree, covered by private/managed care insurance, had at least one paid job, a lower pre-pregnancy BMI, and were more likely to self-identify as a vegetarian than those with low adherence (Table 1). In particular, women with high PHD, HEI, or AHEI adherence had lower baseline physical activity levels than those with low adherence.

When comparing energy, macronutrient, and micronutrient intake across tertiles of adherence to each dietary pattern, energy intakes differed significantly across tertiles of adherence to PHD, DASH, AMED, and HEI. In general, the intake of macronutrients [carbohydrates, protein, and total fat (including monounsaturated, and polyunsaturated and saturated fat)], dietary fiber, and micronutrients (iron, calcium, total folate, Vitamin A, and Vitamin D) differed significantly across tertiles of adherence to each dietary pattern. Notably, women with high adherence to PHD or HEI had lower energy intake, while an opposite trend was observed among women with high adherence to DASH or AMED. Women with high adherence to PHD also had lower intakes of iron, calcium, and Vitamin D, which was a pattern not observed among women with high adherence to DASH, AMED, HEI, or AHEI.

We observed differences in the distribution of women with adequate total and first, second, and third trimester-specific GWG across the tertiles of adherence to each dietary pattern. Notably, women with high adherence to DASH constituted the majority of individuals with adequate total and trimester-specific gestational weight gain (GWG) for all three trimesters. In addition, women with high adherence to AHEI made up the largest proportion of all women with adequate first, second, and third trimester-specific, but not total GWG. The median (p25, p75) total GWG was comparable between women with pre-pregnancy BMI in the normal and overweight range [normal: 12.5 (9.4, 15.6) kg; overweight: 12.7 (8.2, 17.3) kg] and slightly lower for women with pre-pregnancy BMI in the obese range [9.6 (4.7, 15.2) kg].

### 3.2. Adherence to Dietary Patterns and Gestational Weight Gain

Women with high vs. low adherence to DASH, AMED, HEI, or AHEI had 13% to 31% lower odds of inadequate total GWG [ranging from 0.69 (0.48, 0.99) for DASH to 0.87 (0.58, 1.31) for AMED] compared to those with low adherence (Table 2). Null associations were observed between all five dietary patterns and excessive total GWG. No significant association was observed between PHD and GWG, though the point estimates indicated relationships between high PHD adherence and higher odds of both inadequate and excessive total GWG. Stratified analyses by pre-pregnancy BMI revealed similar trends to those of the full cohort, except for the fact that high adherence to DASH was associated with lowered odds of inadequate GWG [0.23 (0.08, 0.63)].

**Figure 1:**
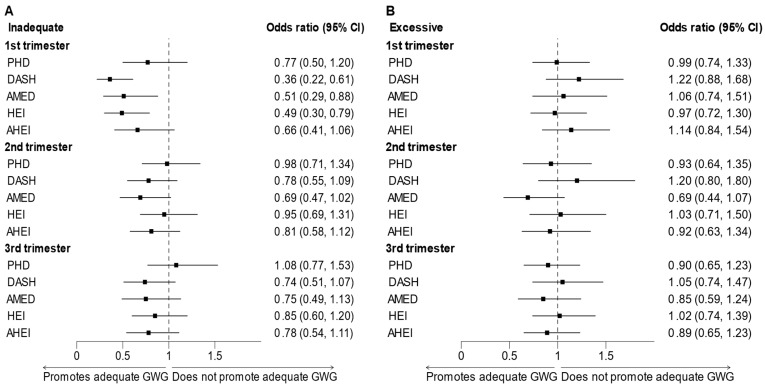
Forest plot of the associations between diet indices and trimester-specific (**A**) inadequate or (**B**) excessive gestational weight gain (adjusted for maternal age, race/ethnicity, education, parity, and total physical activity). Abbreviations: GWG: gestational weight gain.

High adherence to DASH, AMED, or HEI was significantly associated with lower odds of inadequate GWG in the first trimester [DASH: 0.36 (0.22, 0.61), AMED: 0.51 (0.29, 0.88), HEI: 0.49 (0.30, 0.79)] compared to low adherence (Figure 1). Similar relationships were observed between high vs. low adherence to all five dietary patterns and lowered odds of inadequate GWG in the second and third trimesters, although none of these associations reached statistical significance. No significant associations were observed between high adherence to each of the five dietary patterns and excessive GWG in any of the trimesters.

In sensitivity analyses, the odds ratios for total GWG remained largely unchanged after additional adjustment for potential covariates (daily caloric intake or incidence of GDM or HDP) (Supplementary Table S3). Based on the adjusted *p*-values of the associations between each of the five dietary patterns and GWG (total and trimester-specific), the associations between high adherence to DASH or HEI and first-trimester GWG remained significant (Supplementary Table S4). While stratified analyses limited to women without GDM and HDP were consistent with the main analyses, differences were observed when limiting the sample to women with GDM (*N* = 86) or women with HDP (*N* = 105) (Supplementary Table S5). For women with GDM, high adherence to AMED or AHEI lowered the odds of inadequate and excessive total GWG. Similarly, for women with HDP, high adherence to PHD, HEI, or AHEI lowered the odds of inadequate and excessive total GWG.

## 4. Discussion

Healthy diets are widely promoted as advantageous for women who want to become pregnant, as well as throughout pregnancy [32,33]. Among the important expected benefits of adhering to healthy diets is reduced risk of excessive GWG [3,34], which can lead to complications for both the mother and the fetus [35]. Another expected benefit of adhering to healthy diets that tend to be less appreciated is the reduced risk of inadequate GWG [3], which can pose issues for fetal development, obstetric, and perinatal outcomes [36].

We found that high vs. low adherence to the DASH diet, but not to other dietary patterns, significantly decreased the odds of inadequate total GWG. Specifically, high vs. low maternal adherence to two of five dietary patterns (i.e., DASH and HEI) during periconception and early pregnancy was significantly associated with a lower likelihood of inadequate GWG in the first trimester, after correcting for multiple testing. None of the dietary patterns exhibited a significant association with the likelihood of excessive total or trimester-specific GWG. Thus, we inferred that adherence to healthy dietary patterns promotes weight gain to an extent that prevents inadequate GWG but does not increase the odds of excessive GWG.

In this analytical sample of *n* = 1530 pregnant women, the effect sizes for the adjusted odds ratios of inadequate or excessive total and trimester-specific GWG are small based on Cohen’s 1988 criteria [37]. When analyses were limited to women without GDM and HDP, aligning with our main findings, high adherence to all dietary patterns, except PHD, reduced the odds of inadequate total GWG. High adherence to AMED or AHEI reduced the odds of inadequate and excessive total GWG (i.e., promoted adequate GWG) among women with GDM. Among women with HDP, high adherence to PHD, HEI or AHEI reduced the odds of inadequate and excessive total GWG (i.e., promoted adequate GWG). However, these associations were not significant.

### 4.1. Comparison of Findings with Existing Studies

Our findings are generally consistent with those of previous studies that examined the relationships between *a priori* defined dietary patterns and GWG. The existing evidence on the relationship between diet and inadequate GWG has been more consistent. A study on AMED in the United Arab Emirates [38] and another on the Alternate Healthy Eating Index for Pregnancy (AHEI-P) in the US [39] found inverse associations between high adherence to these patterns and inadequate GWG, which aligns with the findings of our study. Meanwhile, existing evidence about the relationship with excessive GWG is mixed. Two studies did not report any significant associations between high vs. low adherence to AHEI-P [39] or HEI-2015 [40] with excessive GWG, aligning with the findings of our study. A recent study on adherence to PHD reported an inverse association with obesity indicators of BMI, waist circumference, and the likelihood of being overweight or obese compared to those with lower adherence [41]. This is unlike our study’s findings, where high vs. low adherence to the PHD was not associated with inadequate or excessive GWG.

In our main analysis, we examined the relationships between each dietary pattern and total GWG. This approach is common among existing studies and was also employed by the 2025 DGAC systematic review committee when studies were selected for inclusion [6]. A shortcoming of our approach is overlooking the possibility that GWG varies by trimester. In addition, the approach does not consider the impact of variation in gestational age at delivery, which means that women have different amounts of time to gain weight during pregnancy [25]. Our study undertook the additional step of examining the relationships between dietary patterns and trimester-specific GWG. We found that high adherence to DASH was associated with reduced odds of inadequate total GWG, primarily driven by GWG in the first trimester, whereas high adherence to AMED or HEI was associated with first-trimester GWG, but not total GWG. These findings make reasonable sense as habitual dietary intake during periconception and early pregnancy—the timing of which our data captured—could be more strongly associated with weight gain in the first trimester than with weight gain in the subsequent trimesters. Maternal adherence to prior dietary patterns does not necessarily persist throughout pregnancy [42], which amplifies the utility of undertaking more nuanced analysis. Examining relationships between dietary patterns and trimester-specific GWG could potentially generate valuable insights that can enable timely dietary interventions during pregnancy to promote adequate GWG [43].

In our stratified analyses, high adherence to AHEI promoted adequate total GWG for women with either GDM or HDP. These results are consistent with findings from non-pregnant, healthy populations on the health benefits of adhering to the AHEI [14]. Taken together, our findings emphasize the importance of examining multiple dietary patterns within a single study as well as within subgroups of women with and without maternal pregnancy complications (GDM or HDP).

### 4.2. Potential Biological Mechanisms

Several potential mechanisms substantiate the biological plausibility of the association between high adherence to DASH or HEI and reduced odds of inadequate GWG. In our study, DASH and HEI scores exhibited the strongest correlation with the consumption of milk and yogurt. This finding corroborates a previous study in which 10 out of 18 plasma metabolomic biomarkers linked to DASH scores were also associated with dairy consumption [44]. Dairy products tend to have high concentrations of lipophilic polychlorinated biphenyls (PCBs) and dioxins, which were found to be associated with an increased risk of obesity among Spanish university graduates [45], a higher BMI, waist circumference, and prevalence of obesity among Spanish middle-aged men and women [46], and a weight gain of more than 10 kg among middle-aged French women [47]. As such, dairy products consumed as part of the DASH and HEI dietary patterns may have contributed to our finding regarding the reduced odds of inadequate GWG by promoting adequate weight gain.

### 4.3. Strengths and Limitations

To our knowledge, this article is the first to examine multiple dietary patterns (including the Planetary Health Diet) during periconception and early pregnancy in the context of a single study with total and trimester-specific GWG. The prospective design of the cohort study enabled us to examine temporal associations between each dietary pattern measured at baseline and subsequent GWG. Wherever possible, we accounted for covariates identified by the 2025 DGAC as key confounders based on their expertise in nutrition and health research [6]. Among the limitations of this study, the FFQ is prone to measurement errors and recall bias [48]. We did not have the ability to verify self-reported dietary consumption as would be required to establish the degree of adherence needed for each dietary pattern. In addition, we lack detailed data on biomarkers [49], which would be necessary to explore and pinpoint mechanisms linking dietary patterns to GWG outcomes. Another missing piece of our study is that we did not consider nutrient intake from dietary supplements due to the lack of granularity in the data collected (i.e., the absolute amounts of individual micronutrients present in the dietary supplement consumed were not always available) [19]. Finally, residual confounding from unmeasured socioeconomic factors (e.g., neighborhood deprivation) cannot be ruled out, though we sought to be comprehensive in considering relevant covariates and utilized a DAG to decide on the minimal number of covariates to include in each model.

## 5. Conclusions

In this cohort of singleton pregnancies, high vs. low adherence to DASH, but not the other dietary patterns, significantly reduced the odds of inadequate total GWG, largely driven by first-trimester GWG. Additionally, high vs. low maternal adherence to DASH or HEI during periconception and early pregnancy was significantly associated with a lower likelihood of inadequate GWG in the first trimester. We did not observe any significant association between dietary patterns and excessive total GWG. This work emphasizes the importance of evaluating (1) trimester-specific GWG, instead of total GWG, to avoid overlooking significant associations between dietary patterns and first-trimester GWG and (2) including multiple dietary patterns in a single study to determine the optimum diet for each subgroup of pregnant women. Our findings provide evidence on the possible dietary periconception and early pregnancy dietary interventions to promote healthy weight gain among women who experience inadequate first-trimester GWG.

Future epidemiological studies involving populations of pregnant women within and outside the US are warranted to confirm our findings. In addition, mechanistic studies on objective measures of diet (e.g., endogenous metabolites, exogenous environmental chemicals, biomarkers related to glucose metabolism and cardiometabolic biomarkers) could advance our understanding of the relationships between dietary patterns and GWG. Given the difficulty in achieving healthy weight gain during pregnancy, this work demonstrates the promising role of dietary patterns, including the PHD, during periconceptional and early pregnancy.

## Figures and Tables

**Table 1:** Characteristics of the study population with high vs. low adherence to five dietary patterns.

Characteristic ^1,3^	PHD		DASH		AMED		HEI		AHEI	
	Low	High	Low	High	Low	High	Low	High	Low	High
N	510	510	443	443	340	322	510	510	510	510
Age (years)										
Mean (SD)	26.3 (5.5)	29.6 (5.2) ^2^	25.9 (5.5)	30.0 (4.9) ^2^	26.5 (5.7)	30.0 (5.3) ^2^	25.8 (5.4)	30.1 (4.9) ^2^	25.8 (5.4)	30.5 (4.7) ^2^
Currently paid jobs										
None	164 (32%)	142 (28%)	153 (35%)	123 (28%)	115 (34%)	91 (28%)	162 (32%)	151 (30%)	153 (30%)	154 (30%)
At least one paid job	346 (68%)	368 (72%)	290 (65%)	320 (72%)	225 (66%)	231 (72%)	348 (68%)	359 (70%)	357 (70%)	356 (70%)
Diet-based vegetarianism (FFQ)										
Full vegetarian (lacto-ovo-, pesco-, and semi-vegetarians)	63 (12%)	150 (30%) ^2^	86 (19%)	96 (22%)	104 (31%)	47 (15%) ^2^	116 (23%)	110 (22%)	121 (24%)	102 (20%)
Non-vegetarian	447 (88%)	356 (70%)	357 (81%)	345 (78%)	236 (69%)	274 (85%)	392 (77%)	398 (78%)	387 (76%)	407 (80%)
Education										
Education less than high school-level	73 (14%)	29 (6%) ^2^	66 (15%)	34 (8%) ^2^	48 (14%)	27 (8%) ^2^	66 (13%)	46 (9%) ^2^	60 (12%)	44 (9%) ^2^
High school diploma or equivalent	142 (28%)	73 (15%)	129 (29%)	62 (14%)	92 (27%)	41 (13%)	142 (28%)	70 (14%)	144 (28%)	64 (13%)
Some college/associate degree	179 (35%)	144 (28%)	157 (35%)	110 (25%)	120 (35%)	83 (26%)	190 (37%)	133 (26%)	195 (38%)	117 (23%)
Undergraduate/postgraduate degree	116 (23%)	264 (52%)	91 (21%)	237 (53%)	80 (24%)	170 (53%)	112 (22%)	261 (51%)	111 (22%)	285 (56%)
Health insurance										
Private/managed care	248 (49%)	375 (74%) ^2^	210 (47%)	313 (71%) ^2^	184 (54%)	232 (72%) ^2^	242 (47%)	357 (70%) ^2^	250 (49%)	377 (74%) ^2^
Other/Medicaid/Self-paid	262 (51%)	135 (26%)	233 (53%)	130 (29%)	156 (46%)	90 (28%)	268 (53%)	153 (30%)	260 (51%)	133 (26%)
Marital status										
Married or living with a partner	315 (62%)	414 (81%) ^2^	259 (59%)	373 (84%) ^2^	225 (66%)	257 (80%) ^2^	302 (59%)	430 (84%) ^2^	299 (59%)	443 (87%) ^2^
Not married	194 (38%)	96 (19%)	183 (41%)	70 (16%)	114 (34%)	65 (20%)	207 (41%)	80 (16%)	210 (41%)	67 (13%)
Race/ethnicity										
Non-Hispanic White	65 (13%)	160 (31%) ^2^	52 (12%)	154 (35%) ^2^	72 (21%)	81 (25%) ^2^	73 (14%)	135 (26%) ^2^	88 (17%)	137 (27%) ^2^
Non-Hispanic Black	241 (47%)	80 (16%)	221 (50%)	66 (15%)	134 (39%)	74 (23%)	257 (50%)	84 (16%)	253 (50%)	73 (14%)
Hispanic	144 (28%)	138 (27%)	102 (23%)	135 (30%)	96 (28%)	83 (26%)	119 (23%)	165 (32%)	125 (25%)	140 (27%)
Asian/Pacific Islander	60 (12%)	132 (26%)	68 (15%)	88 (20%)	38 (11%)	84 (26%)	61 (12%)	126 (25%)	44 (9%)	160 (31%)
Parity (number of births)										
0	218 (43%)	251 (49%) ^2^	200 (45%)	211 (48%)	144 (42%)	157 (49%)	245 (48%)	239 (47%)	238 (47%)	232 (45%)
1	178 (35%)	183 (36%)	150 (34%)	159 (36%)	130 (38%)	114 (35%)	165 (32%)	180 (35%)	174 (34%)	189 (37%)
2 or more	114 (22%)	76 (15%)	93 (21%)	73 (16%)	66 (19%)	51 (16%)	100 (20%)	91 (18%)	98 (19%)	89 (17%)
Pre-pregnancy BMI (kg/m^2^)										
Mean (SD)	25.9 (5.3)	24.4 (4.6) ^2^	25.8 (5.4)	24.6 (4.4)^2^	26.2 (5.2)	24.0 (4.1) ^2^	25.8 (5.3)	24.7 (4.6) ^2^	26.0 (5.3)	24.4 (4.4) ^2^
19 to <25.0 (normal)	272 (53%)	337 (66%) ^2^	233 (53%)	286 (65%) ^2^	167 (49%)	222 (69%) ^2^	271 (53%)	325 (64%) ^2^	258 (51%)	338 (66%) ^2^
≥25.0 to <30.0 (overweight)	142 (28%)	112 (22%)	122 (28%)	109 (25%)	97 (29%)	76 (24%)	142 (28%)	127 (25%)	151 (30%)	116 (23%)
≥30.0 (obese)	96 (19%)	61 (12%)	88 (20%)	48 (11%)	76 (22%)	24 (7%)	97 (19%)	58 (11%)	101 (20%)	56 (11%)
Self-defined vegetarianism (FFQ)										
Non-vegetarian	485 (97%)	451 (91%) ^2^	423 (98%)	379 (88%) ^2^	327 (98%)	283 (89%) ^2^	484 (97%)	452 (90%) ^2^	481 (97%)	454 (90%) ^2^
Vegetarian	16 (3%)	45 (9%)	9 (2%)	54 (12%)	5 (2%)	35 (11%)	16 (3%)	48 (10%)	17 (3%)	50 (10%)
Total physical activity (MET-min/week)										
Median (p25, p75)	312 (209, 418)	274 (212, 378) ^2^	295 (204, 388)	292 (225, 394)	295 (201, 416)	294 (225, 400)	304 (205, 417)	279 (214, 384)	306 (206, 407)	275 (214, 378)
Energy (kcal/day), mean (SD)	2595 (1224)	1846 (730) ^2^	2182 (1122)	2271 (919) ^2^	1661 (804)	2554 (989) ^2^	2361 (1185)	2006 (837) ^2^	2169 (1158)	2121 (864)
Macronutrients, mean (SD)										
Carbohydrates (g/day),	342.6 (190.9)	253.2 (112.4) ^2^	291.9 (183.4)	312.1 (134.1) ^2^	224 (132)	338 (142) ^2^	323.3 (190.7)	268.7 (123.9) ^2^	303.1 (190.0)	275.1 (125.1) ^2^
Protein (g/day),	101.3 (48.1)	70.6 (30.1) ^2^	78.1 (38.8)	93.5 (41.6) ^2^	59.9 (26.6)	107.0 (45.0) ^2^	82.9 (43.5)	84.3 (38.3)	75.3 (40.1)	90.7 (39.7) ^2^
Total fat (g/day),	95.6 (49.0)	66.6 (30.5) ^2^	81.5 (44.0)	79.0 (35.4) ^2^	61.4 (33.0)	92.6 (40.8) ^2^	85.7 (46.9)	72.0 (32.4) ^2^	76.7 (44.5)	78.9 (34.2)
Monounsaturated fat (g/day),	36.0 (18.9)	26.6 (13.3) ^2^	30.9 (16.8)	31.0 (14.5) ^2^	22.6 (12.2)	37.1 (16.7) ^2^	32.1 (17.8)	29.0 (14.3) ^2^	28.8 (16.9)	31.5 (14.6) ^2^
Polyunsaturated fat (g/day),	19.8 (10.9)	14.7 (8.0) ^2^	17.2 (10.2)	17.0 (7.7)	12.5 (7.8)	20.4 (8.8) ^2^	17.3 (10.2)	16.0 (7.5) ^2^	15.5 (9.8)	17.6 (7.9) ^2^
Saturated fat (g/day),	32.3 (17.2)	19.9 (9.1) ^2^	27.3 (15.1)	24.6 (12.5) ^2^	21.6 (12.0)	27.7 (13.7) ^2^	29.7 (16.9)	21.3 (9.9) ^2^	26.5 (15.7)	23.4 (11.1) ^2^
Dietary fiber (g/day),	22.1 (13.9)	22.9 (11.0)	16.2 (9.0)	29.2 (13.2) ^2^	13.1 (6.8)	31.5 (13.6) ^2^	18.1 (10.8)	25.9 (13.3) ^2^	16.3 (9.6)	26.8 (13.1) ^2^
Micronutrients, mean (SD)										
Iron (mg/day)	19.6 (10.0)	16.8 (7.7) ^2^	15.7 (8.1)	20.9 (9.3) ^2^	12.2 (5.3)	23.4 (9.6) ^2^	17.6 (9.3)	18.3 (8.7)	16.1 (8.7)	19.3 (9.2) ^2^
Calcium (mg/day)	1123.1 (677.9)	876.2 (407.1) ^2^	801.0 (450.0)	1255.8 (666.6) ^2^	732.1 (401.2)	1217.3 (552.7) ^2^	940.4 (591.7)	1049.5 (564.9) ^2^	888.7 (540.6)	1066.6 (565.6) ^2^
Total folate (dietary equivalent) (mcg/day)	678.4 (364.7)	595.7 (277.7) ^2^	543.3 (294.9)	739.2 (346.2) ^2^	430.5 (207.4)	812.2 (351.2) ^2^	612.8 (334.3)	640.2 (314.8)	558.3 (310.0)	678.1 (333.6) ^2^
Vitamin A (mcg of retinol equivalent/day)	1516.6 (1114.8)	1539.1 (998.6)	1055.2 (737.3)	2039.7 (1168.1) ^2^	893.0 (663.2)	2227.8 (1226.4) ^2^	1163.9 (858.9)	1863.2 (1185.1) ^2^	1062.2 (720.3)	1953.0 (1267.2) ^2^
Vitamin D (calciferol) (mcg/day)	7.2 (4.8)	4.5 (2.9) ^2^	4.9 (3.5)	7.1 (5.0) ^2^	4.0 (3.0)	7.5 (4.3) ^2^	5.1 (4.1)	6.3 (4.1) ^2^	4.8 (3.8)	6.6 (4.2) ^2^
Gestational weight gain (kg)										
Total GWG, median (p25, p75)	11.7 (7.7, 15.6)	12.6 (9.4, 16.0) ^2^	11.0 (6.8, 15.3)	12.8 (9.7, 16.6) ^2^	11.5 (7.3, 16.1)	12.9 (9.2, 16.7) ^2^	11.6 (7.7, 15.6)	12.5 (9.5, 16.2) ^2^	11.5 (7.8, 16.1)	12.8 (9.2, 16.3) ^2^
Women with adequate GWG										
Total	158 (32.6%)	171 (35.3%)	128 (26.4%)	156 (32.2%) ^2^	95 (19.6%)	103 (21.2%)	144 (29.7%)	178 (36.7%)	142 (29.3%)	171 (35.3%)
1st trimester	166 (31.0%)	185 (34.6%) ^2^	147 (27.5%)	159 (29.7%) ^2^	107 (20.0%)	117 (21.9%) ^2^	165 (30.8%)	194 (36.3%) ^2^	174 (32.5%)	193 (36.1%) ^2^
2nd trimester	134 (30.1%)	169 (38.0%) ^2^	111 (24.9%)	152 (34.2%) ^2^	76 (17.1%)	108 (24.3%) ^2^	136 (30.6%)	169 (38.0%)	123 (27.6%)	168 (37.8%) ^2^
3rd trimester	186 (30.9%)	222 (36.9%)	158 (26.3%)	191 (31.8%) ^2^	113 (18.8%)	139 (23.1%)	186 (30.9%)	223 (37.1%)	172 (28.6%)	228 (37.9%) ^2^

Abbreviations: PHD: Planetary Health Diet; DASH: Dietary Approaches to Stop Hypertension; AMED: Alternative Mediterranean Diet; HEI: Healthy Eating Index-2010; AHEI: Alternate Healthy Eating Index-2010; GWG: gestational weight gain. ^1^ *n*/*N* (% by columns) for categorical variables and means (SDs) for continuous variables unless otherwise stated. ^2^ Significant *p*-values (*p* < 0.05) for Pearson’s chi-square test with categorical variables or one-way Analysis of Variance (ANOVA) or Kruskal–Wallis test with continuous variables to compare across tertiles of each dietary pattern. ^3^ Overall (*n* = 1530): missing data on marital status (*n* = 1); self-defined vegetarianism (based on the FFQ) (*n* = 35); diet-based vegetarianism (based on the FFQ) (*n* = 4).

**Table 2:** Association between gestational weight gain and adherence to five dietary patterns during periconception and early pregnancy.

Diet Index	Odds Ratios (95% Confidence Intervals) for Tertile 3 (High) vs. Tertile 1 (Low) ^1^	
	Inadequate vs. Adequate GWG	Excessive vs. Adequate GWG
Full cohort (*N* = 1530)		
PHD	1.11 (0.80, 1.55)	1.06 (0.76, 1.47)
DASH	0.69 (0.48, 0.99) ^2^	1.17 (0.82, 1.67)
AMED	0.87 (0.58, 1.31)	1.10 (0.74, 1.62)
HEI	0.78 (0.56, 1.10)	0.95 (0.68, 1.32)
AHEI	0.81 (0.57, 1.15)	1.09 (0.78, 1.52)
Normal weight (*N* = 881)		
PHD	1.17 (0.78, 1.76)	1.13 (0.70, 1.84)
DASH	0.83 (0.53, 1.30)	1.20 (0.70, 2.05)
AMED	0.87 (0.53, 1.43)	1.14 (0.65, 1.99)
HEI	0.81 (0.53, 1.24)	1.00 (0.61, 1.62)
AHEI	0.82 (0.53, 1.27)	1.14 (0.69, 1.87)
Overweight (*N* = 403)		
PHD	1.13 (0.48, 2.69)	1.52 (0.83, 2.81)
DASH	0.23 (0.08, 0.63) ^2^	1.01 (0.53, 1.91)
AMED	0.91 (0.30, 2.70)	1.67 (0.78, 3.56)
HEI	0.45 (0.18, 1.10)	0.93 (0.51, 1.72)
AHEI	0.61 (0.24, 1.55)	1.25 (0.67, 2.30)
Obese (*N* = 246)		
PHD	0.69 (0.25, 1.91)	0.85 (0.35, 2.04)
DASH	0.68 (0.20, 2.25)	2.82 (1.02, 7.82) ^2^
AMED	0.68 (0.16, 2.94)	1.33 (0.38, 4.66)
HEI	0.94 (0.34, 2.60)	1.25 (0.52, 3.01)
AHEI	0.57 (0.20, 1.61)	1.11 (0.46, 2.67)

Abbreviations: GWG: gestational weight gain. ^1^ Adjusted for maternal age, race/ethnicity, education, parity, and total physical activity. ^2^ *p* < 0.05.

## Data Availability

The original contributions presented in this study are included in the article/Supplementary Material. Further inquiries can be directed to the corresponding author.

## Supplementary Materials

The following supporting information can be downloaded at https://www.mdpi.com/article/10.3390/nu17162707/s1, Figure S1: Flowchart of analytical sample. Supplementary Figure S2: Spearman correlations between dietary pattern scores. Figure S3: Spearman correlations between dietary pattern scores and food groups. Figure S4: Directed Acyclic Graph (DAG) to examine the associations between dietary patterns and gestational weight gain. Table S1: Cut-offs and number of women in each tertile of each dietary pattern. Table S2: Comparison of baseline characteristics between included and excluded women from the analytical sample. Table S3: Association between 5 healthy dietary patterns during periconception and early pregnancy (comparing high vs. low adherence) and total gestational weight gain, with additional adjustment for covariates. Table S4: Unadjusted and adjusted *p*-values of key associations presented in Table 1 and Figure 1. Table S5: Association between 5 healthy dietary patterns during periconception and early pregnancy (comparing high vs. low adherence) and total gestational weight gain in subgroups of pregnant women (1) without gestational diabetes and hypertensive disorders of pregnancy; (2) gestational diabetes; and (3) hypertensive disorders of pregnancy. Supplementary Text: Supplementary text for Figures S2 and S3—Correlations between dietary pattern scores and food groups.

## Attestation

Data regarding any of the subjects in this study have not been previously published unless specified. The manuscript content was cleared for publication by the NICHD, but the study sponsor played no role in the design or conduct of the study; the collection, management, analysis, or interpretation of the data; the preparation or review of the manuscript; or the decision to submit the manuscript for publication.

## Abbreviations

The following abbreviations are used in this manuscript:

AHEI	Alternate Healthy Eating Index-2010
AHEI-P	Alternate Healthy Eating Index for Pregnancy
AMED	Alternative Mediterranean diet
ASA24	Automated Self-Administered 24-Hour Dietary Assessment Tool
BMI	Body Mass Index
DAG	Directed Acyclic Graph
DASH	Dietary Approaches to Stop Hypertension
DGA	Dietary Guidelines for Americans
DGAC	Dietary Guidelines Advisory Committee
DHQ-II	Diet History Questionnaire-II
HEI	Healthy Eating Index-2010
IOM	Institute of Medicine
FFQ	Food-Frequency Questionnaire
GWG	Gestational Weight Gain
MPED	My Pyramid Equivalent Database
NICHD	*Eunice Kennedy Shriver* National Institute of Child health and Human Development
PHD	Planetary Health Diet
US	United States
USDA	United States Department of Agriculture
