# Supplementary material for: Maternal Adherence to Healthy Dietary Patterns During Pregnancy and Gestational Weight Gain
Source: Nutrients. 2025 Aug 21;17(16):2707. doi: 10.3390/nu17162707 (PMC12389537; doi:10.3390/nu17162707)
Supplement: Supplementary file 1 [file nutrients-17-02707-s001.zip › nutrients-3760631-supplementary.pdf]

Supplementary Material

**Supplemental Material**	**Description**	**Page**
Supplementary Figure S1	Flowchart of analytical sample.	2
Supplementary Figure S2	Spearman correlations between dietary pattern scores.	3
Supplementary Figure S3	Spearman correlations between dietary pattern scores and food groups.	4-5
Supplementary Figure S4	Directed Acyclic Graph (DAG) to examine associations between dietary patterns and gestational weight gain.	6
Supplementary Table S1	Cut-offs and number of women in each tertile of each dietary pattern.	7
Supplementary Table S2	Comparison of baseline characteristics between women included and excluded from the analytical sample.	8-9
Supplementary Table S3	Association between 5 healthy dietary patterns during periconception and early pregnancy. (comparing high vs low adherence) and total gestational weight gain, additional adjustment for covariates.	10
Supplementary Table S4	Unadjusted and adjusted p-values of key associations presented in Table 1 and Figure 1.	11
Supplementary Table S5	Association between 5 healthy dietary patterns during periconception and early pregnancy (comparing high vs low adherence) and total gestational weight gain in subgroups of pregnant women (1) without gestational diabetes and hypertensive disorders of pregnancy, (2) gestational diabetes and (3) hypertensive disorders of pregnancy.	12-13
Supplementary Text	Supplementary Text for Supplementary Figures S2 and S3 - Correlations between dietary pattern scores and food groups	14

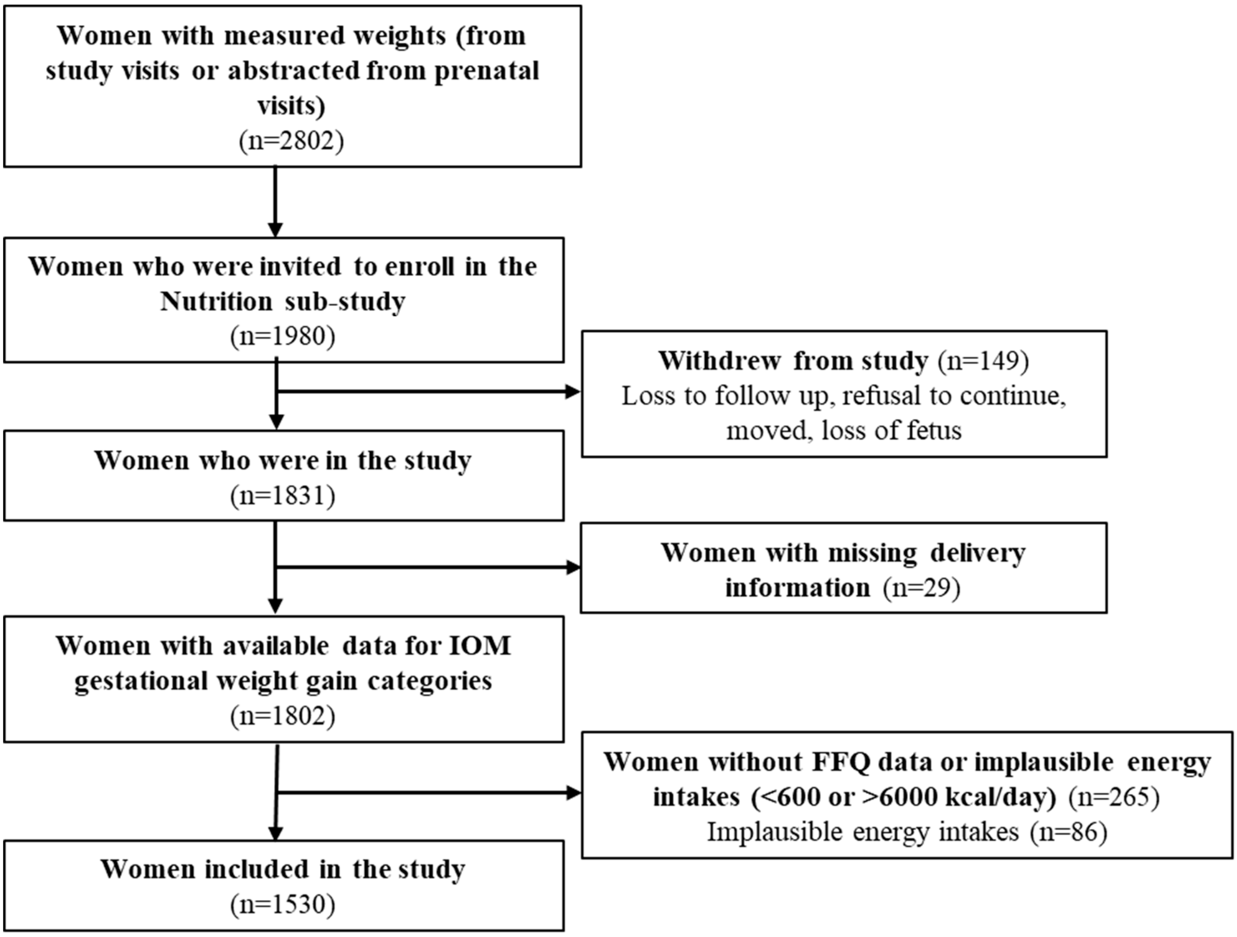

Supplementary Figure S1: Flowchart of analytical sample.

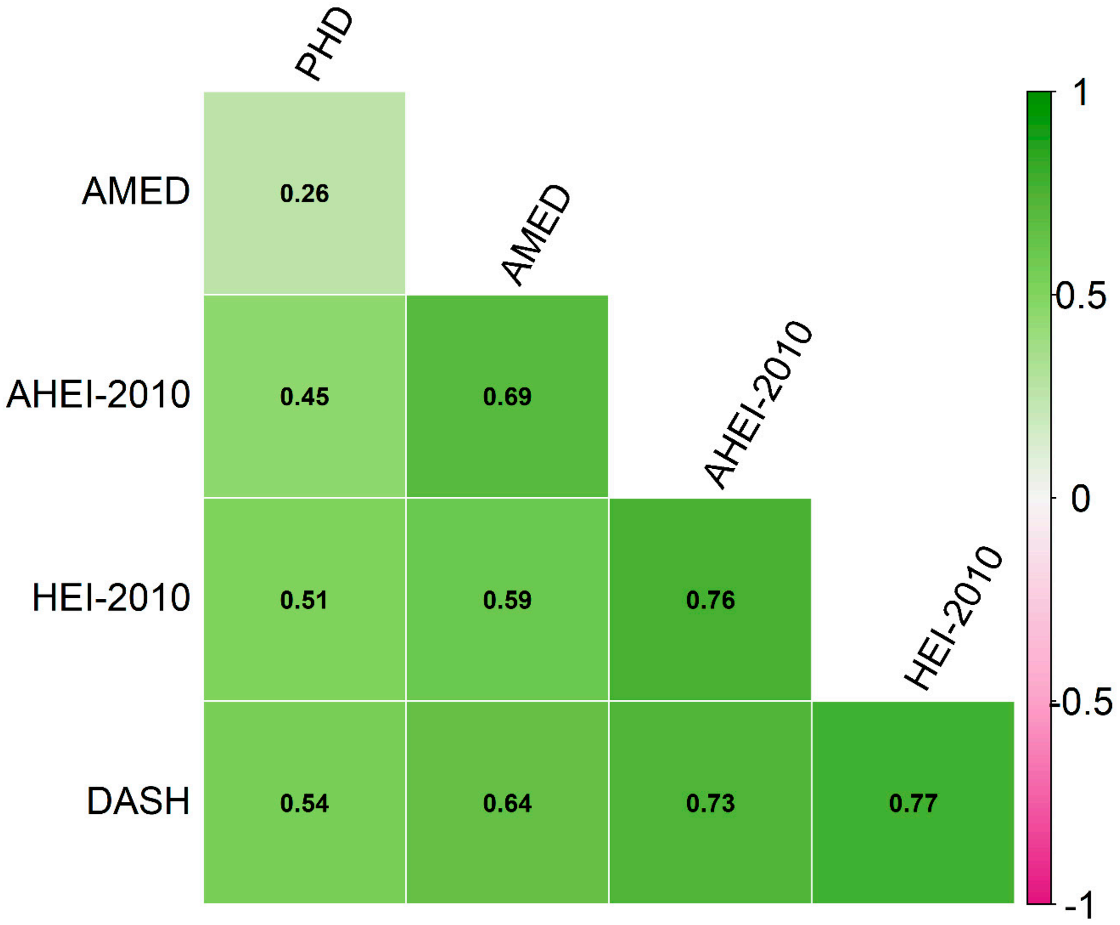

Supplementary Figure S2: Spearman correlations between five dietary pattern scores.

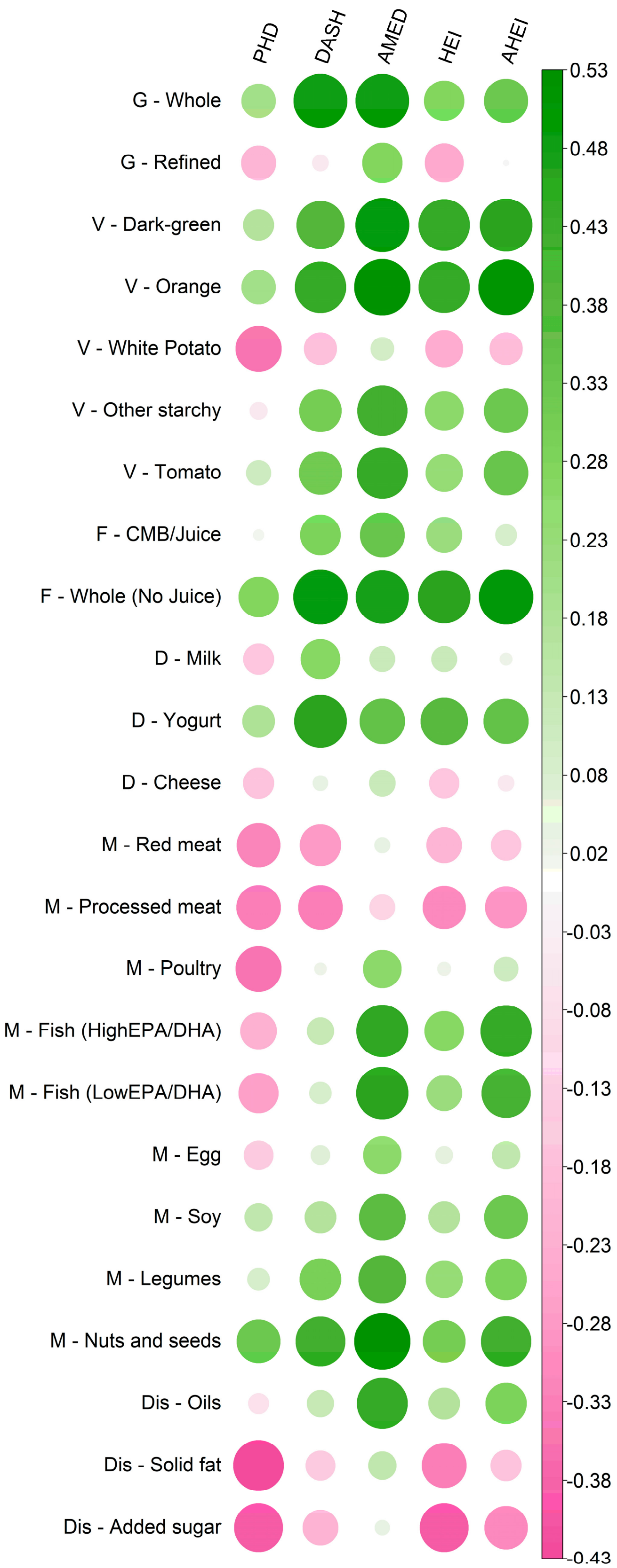
PHD
DASH
AMED
HEI
AHEI
G - Whole
G - Refined
V - Dark-green
V - Orange
V - White Potato
V - Other starchy
V - Tomato
F - CMB/Juice
F - Whole (No Juice)
D - Milk
D - Yogurt
D - Cheese
M - Red meat
M - Processed meat
M - Poultry
M - Fish (HighEPA/DHA)
M - Fish (LowEPA/DHA)
M - Egg
M - Soy
M - Legumes
M - Nuts and seeds
Dis - Oils
Dis - Solid fat
Dis - Added sugar
0.53
0.48
0.43
0.38
0.33
0.28
0.23
0.18
0.13
0.08
0.02
-0.03
-0.08
-0.13
-0.18
-0.23
-0.28
-0.33
-0.38
-0.43

Supplementary Figure S3: Spearman correlations between dietary pattern scores and food groups. Legend: *G - Whole*: Grains (Whole Grains), *G – Refined*: Grains (Refined grains), *V - Dark-green*: Vegetables (Dark green), *V – Orange*: Vegetables (Orange), *V - White Potato*: Vegetables (Potato), *V - Other starchy:* V- Vegetables (Other starchy), *V - Tomato:* V- Vegetables (Tomato), *F - CMB/Juice*: Fruit: citrus fruits, melons, berries, and their juices, *F - Whole (No Juice)*: Fruit (Whole fruit), *D – Milk*: Dairy (Milk), *D – Yogurt*: Dairy (Yogurt), *D – Cheese*: Dairy (Cheese)*, M - Red meat*: Meat (beef, pork, veal, lamb, and game), *M - Processed meat:* Meat (frankfurters, sausages, and luncheon meats)*, M – Poultry*: Meat (chicken, turkey, and other poultry), *M - Fish (HighEPA/DHA): Meat (Fish, shellfish, and other seafood that are high in the n-3 fatty acids eicosapentaenoic acid (EPA) and docosahexaenoic acid (DHA)), M - Fish (LowEPA/DHA): Meat (Fish, shellfish, and other seafood that are low in the n-3 fatty acids eicosapentaenoic acid (EPA) and docosahexaenoic acid (DHA)), M – Egg: Meat (Eggs and egg substitutes)*, *M – Soy: Meat (soybean products)*, *M – Legumes: Meat (cooked dry beans and peas)*, *M - Nuts and seeds: Meat (Nuts and seeds)*, *Dis – Oils*: Discretionary oils, *Dis - Solid fat*: Discretionary solid fat*, Dis - Added sugar: Added sugars.*

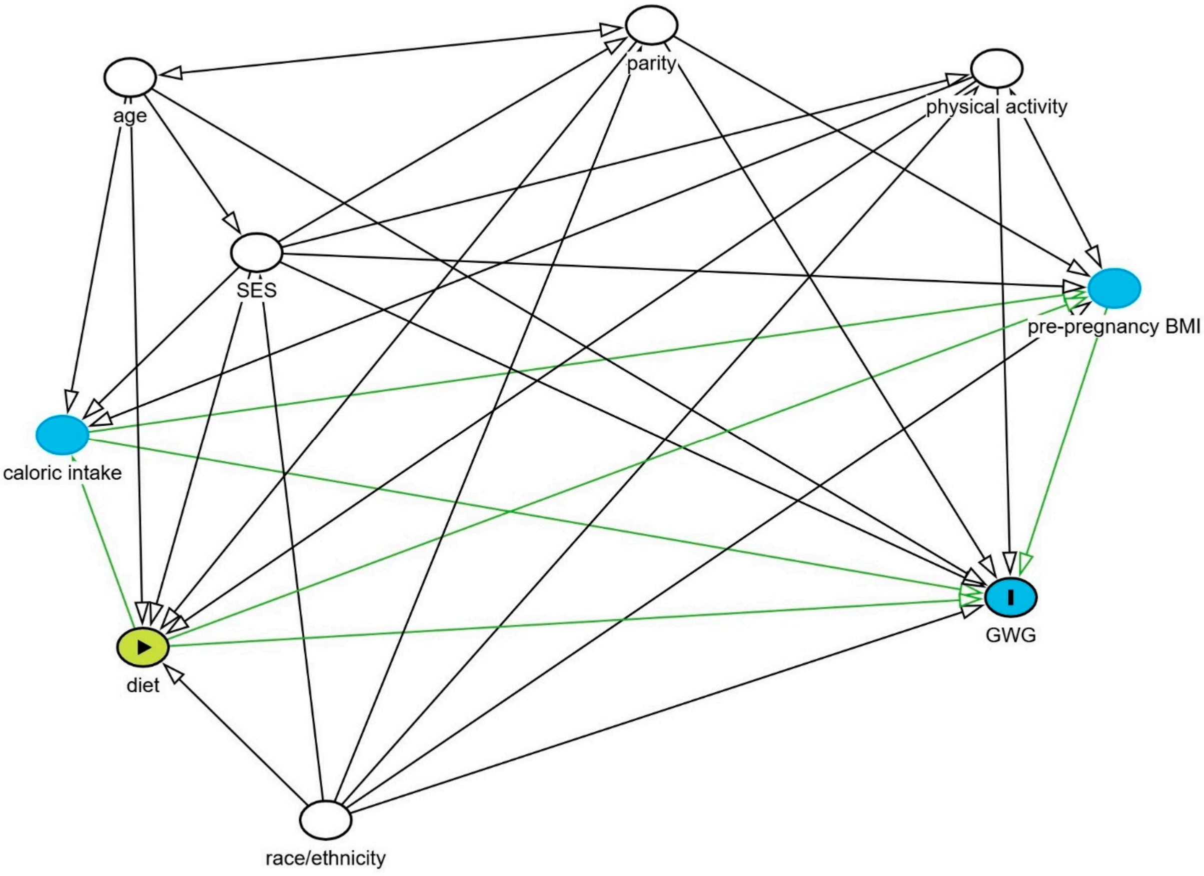

Supplementary Figure S4: Directed Acyclic Graph (DAG) to examine the associations between dietary patterns and gestational weight gain.

Supplementary Table S1: Cut-offs and number of women in each tertile of each dietary pattern.

	Theoretical range	T1 (Low)	T2 (Moderate)	T3 (High)
**PHD**	0 to 140	< 90	90 ≤ PHD score ≤ 99	> 99
**N**		510	510	510
**DASH**	0 to 40	< 22	22 ≤ DASH score ≤ 26	> 26
**N**		443	644	443
**AMED**	0 to 9	< 3	3 ≤ AMED score ≤ 5	> 5
**N**		340	868	322
**HEI-2010**	0 to 100	< 61	61 ≤ HEI-2010 score ≤ 70	> 70
**N**		510	510	510
**AHEI-2010**	0 to 110	< 48	48 ≤ AHEI-2010 score ≤ 57	> 57
**N**		510	510	510

Supplementary Table S2: Comparison of baseline characteristics between included and excluded women from the analytical sample.

Characteristic	Included	Excluded
N	1530	450
Age (years)		
Mean (SD)	28.1 (5.6) [2]	27.1 (5.6)
Race/ethnicity		
Non-Hispanic White	318 (21%) [2]	48 (11%)
Non-Hispanic Black	458 (30%)	179 (40%)
Hispanic	462 (30%)	126 (28%)
Asian/Pacific Islander	292 (19%)	97 (22%)
Marital status		
Married or living with a partner	1,119 (73%) [2]	285 (63%)
Not married	410 (27%)	165 (37%)
Education		
Less than High school	172 (11%) [2]	89 (20%)
High school diploma or equivalent	304 (20%)	96 (21%)
Some college/ Associate degree	476 (31%)	150 (33%)
Undergraduate/Postgraduate degree	578 (38%)	115 (26%)
Currently paid jobs		
None	472 (31%) [2]	175 (39%)
At least one paid job	1,058 (69%)	275 (61%)
Health insurance		
Private/managed care	916 (60%) [2]	140 (47%)
Others/Medicaid/Self-pay	614 (40%)	161 (53%)
Parity (Number of births)		
0	694 (45%)	197 (44%)
1	536 (35%)	152 (34%)
2 or more	300 (20%)	101 (22%)
Pre-pregnancy BMI (kg/m^2)		
Mean (SD)	28.1 (5.6)	27.1 (5.6)
19 to < 25.0 (Normal)	881 (58%)	251 (56%)
≥ 25.0 to < 30.0 (Overweight)	403 (26%)	134 (30%)
≥ 30.0 (Obese)	246 (16%)	65 (14%)
Total physical activity (MET-min/ week)		
Median (p25, p75)	288 (209, 392)	282 (194, 389)

Characteristic (continued).	**Included**	**Excluded**
Self-defined vegetarianism (FFQ)		
Non vegetarian	1,409 (94%)	140 (92%)
Vegetarian	86 (6%)	13 (8%)
Diet-based vegetarianism (FFQ)		
Full vegetarian (Lacto-ovo-, Pesco- and Semi-vegetarian)	327 (21%)	16 (19%)
Non vegetarian	1,199 (79%)	68 (81%)

[1] n / N (%) for categorical variables and mean (SD) for continuous variables unless otherwise stated.

[2] Significant p values ($p < 0.05$) for Pearson's chi-square test for categorical variables or one-way Analysis of Variance (ANOVA) for continuous variables comparing between included and excluded women.

[3] Overall (n=1980): Missing data for marital status (n=1), health insurance (n=149), self-defined vegetarianism (based on the FFQ) (n = 332), Diet-based vegetarianism (based on the FFQ) (n =370).

Supplementary Table S3: Association between 5 healthy dietary patterns during periconception and early pregnancy (comparing high vs low adherence) and total gestational weight gain, additional adjustment for covariates.

**Diet index**	**Odds ratios (95% confidence intervals) for T3 (High) vs. T1 (Low)**			
	**Inadequate vs Adequate**		**Excessive vs Adequate**	
	**Model 1 [1]**	**Model 2 [2]**	**Model 1 [1]**	**Model 2 [2]**
PHD	1.10 (0.92, 1.32)	1.11 (0.79, 1.55)	1.07 (0.88, 1.30)	1.09 (0.78, 1.51)
DASH	*0.69 (0.59, 0.81)* [3]	*0.69 (0.48, 0.99)* [3]	1.17 (1.01, 1.36)	1.19 (0.83, 1.70)
AMED	*0.87 (0.77, 0.99)* [3]	0.87 (0.58, 1.31)	1.12 (0.99, 1.26)	1.13 (0.76, 1.67)
HEI	*0.78 (0.63, 0.96)* [3]	0.78 (0.56, 1.10)	0.95 (0.77, 1.17)	0.97 (0.70, 1.35)
AHEI	*0.82 (0.70, 0.95)* [3]	0.81 (0.57, 1.15)	1.09 (0.95, 1.24)	1.12 (0.80, 1.56)

[1] Model 1: Adjusted for maternal age, race/ethnicity, education, parity, physical activity, *daily caloric intake*

[2] Model 2: Adjusted for maternal age, race/ethnicity, education, parity, physical activity*, having gestational diabetes mellitus or hypertensive disorders of pregnancy in the current pregnancy*

[3] $p < 0.05$ are in italics

Supplementary Table S4: Unadjusted and adjusted p-values of key associations presented in Table 1 and Figure 1.

Diet index	Odds ratios (95% confidence intervals) for Tertile 3 (High) vs. Tertile 1 (Low)			
	Inadequate vs. Adequate GWG		Excessive vs. Adequate GWG	
	Unadjusted p value	Adjusted p-value (Benjamini-Hochberg)	Unadjusted p value	Adjusted p-value (Benjamini-Hochberg)
**Full cohort (N=1530) – Total GWG**				
PHD	5.32E-01	6.65E-01	7.24E-01	9.03E-01
DASH	**4.33E-02**	2.16E-01	3.91E-01	9.03E-01
AMED	5.03E-01	6.65E-01	6.36E-01	9.03E-01
HEI	1.59E-01	3.54E-01	7.67E-01	9.03E-01
AHEI	2.45E-01	3.81E-01	6.25E-01	9.03E-01
**Full cohort (N=1530) – 1st trimester GWG**				
PHD	2.46E-01	3.81E-01	9.54E-01	9.54E-01
DASH	**1.09E-04**	**2.17E-03**	2.30E-01	9.03E-01
AMED	**1.48E-02**	9.87E-02	7.46E-01	9.03E-01
HEI	**3.23E-03**	**3.23E-02**	8.20E-01	9.12E-01
AHEI	8.74E-02	2.50E-01	3.94E-01	9.03E-01
**Full cohort (N=1530) – 2nd trimester GWG**				
PHD	9.00E-01	9.00E-01	7.15E-01	9.03E-01
DASH	1.47E-01	3.54E-01	3.84E-01	9.03E-01
AMED	6.49E-02	2.50E-01	9.96E-02	9.03E-01
HEI	7.65E-01	8.50E-01	8.77E-01	9.23E-01
AHEI	2.03E-01	3.81E-01	6.59E-01	9.03E-01
**Full cohort (N=1530) – 3rd trimester GWG**				
PHD	7.35E-01	8.50E-01	4.87E-01	9.03E-01
DASH	2.48E-01	3.81E-01	4.55E-01	9.03E-01
AMED	7.72E-02	2.50E-01	3.84E-01	9.03E-01
HEI	8.92E-01	9.00E-01	2.29E-01	9.03E-01
AHEI	4.85E-01	6.65E-01	3.18E-01	9.03E-01

Values in bold text are statistically significant ($p < 0.05$ or 5.00E-02)

Supplementary Table S5: Association between 5 healthy dietary patterns during periconception and early pregnancy (comparing high vs low adherence) and total gestational weight gain, by subgroups of pregnant women (1) without gestational diabetes and hypertensive disorders of pregnancy, (2) gestational diabetes and (3) hypertensive disorders of pregnancy.

**Diet index**	**Odds ratios (95% confidence intervals) for Tertile 3 (High) vs. Tertile 1 (Low)**	
	**Inadequate vs. Adequate GWG**	**Excessive vs. Adequate GWG**
**Women without gestational diabetes and hypertensive disorders of pregnancy (N=1348)**		
PHD	1.11 (0.78, 1.57)	1.16 (0.82, 1.65)
DASH	0.69 (0.47, 1.01)	1.18 (0.80, 1.72)
AMED	0.85 (0.55, 1.31)	1.13 (0.75, 1.72)
HEI	0.79 (0.55, 1.13)	0.96 (0.67, 1.36)
AHEI	0.86 (0.59, 1.25)	1.16 (0.80, 1.66)
**Women with gestational diabetes (N=86)**		
PHD	1.16 (0.17, 7.91)	0.98 (0.19, 4.96)
DASH	1.20 (0.13, 10.8)	1.10 (0.19, 6.29)
AMED	0.88 (0.08, 10.3)	0.90 (0.11, 7.28)
HEI	1.95 (0.27, 14.2)	1.15 (0.22, 5.96)
AHEI	0.94 (0.13, 6.70)	0.62 (0.12, 3.25)
**Women with hypertensive disorders of pregnancy (N=105)**		
PHD	0.23 (0.03, 1.96)	0.23 (0.05, 1.17)
DASH	0.30 (0.04, 2.53)	1.00 (0.18, 5.65)

AMED	1.06 (0.17, 6.80)	0.33 (0.05, 2.26)
HEI	0.41 (0.06, 2.70)	0.85 (0.19, 3.69)
AHEI	0.15 (0.02, 1.40)	0.69 (0.15, 3.19)

Abbreviations: GWG: gestational weight gain.

[1] Adjusted for maternal age, race/ethnicity, education, parity, total physical activity.

[2] $p < 0.05$ are in italics

Supplementary Text for Supplementary Figures S2 and S3

Correlations between dietary pattern scores and food groups

The Spearman correlation coefficients between the dietary pattern scores ranged from 0.26 (AMED and PHD) to 0.77 (DASH and HEI) **(Supplementary Figure S2)**. Comparing the correlations between individual food groups and the dietary pattern scores, all dietary pattern scores were positively correlated with whole grains, dark green and orange vegetables, tomato, fruits (including fruit juice), yogurt, nuts and seeds, soy and legumes but negatively correlated with white potatoes, red meat, processed meat, solid fat and added sugars, except for AMED **(Supplementary Figure S3)**. Notably, AMED scores were positively correlated with refined grains and oils, DASH scores were positively correlated with milk and yogurt, and PHD was negatively correlated with animal-sourced foods such as red and processed meat, poultry, fish and eggs.
